# Characteristics, causes and impact of headache among a sample of physicians working during COVID-19 pandemic

**DOI:** 10.1186/s41983-022-00520-7

**Published:** 2022-07-14

**Authors:** Sherien Farag, Ahmed ElSadek, Waleed Salah-Eldin, Shady Samy Georgy, Mai Fathy

**Affiliations:** 1 Neurology Department, Ain Shams University, Cairo, Egypt; 2 Community Medicine Department, Ain Shams University, Cairo, Egypt

**Keywords:** Personal protective equipment, External compression headache, Tension headache, Post COVID-19 headache

## Abstract

**Background:**

Headache is considered a common health problem affecting physicians during Coronavirus disease-19 (COVID-19) pandemic and has direct impact on their productivity. Wearing personal protective equipment (PPE), stress and lack of sleep are common factors affecting their headache.

**Results:**

Out of 165 participants, 38(23%) experienced new onset headache. Participants using Combined Face and eye PPE usage were at higher risk of developing headache compared to single PPE users, Participants wearing face shield were at higher risk of developing headache compared to eyewear non users.

**Conclusion:**

COVID-19 hospital’s physicians may experience new-onset headache or change in their previously existing headache, mostly disposed by PPE eyewear and combined face and eye PPE.

## Background

The massive spread of Coronavirus disease-19 (COVID-19), which started in Wuhan, led to its characterization as a pandemic by World Health Organization (WHO) on March 2019 [1]. Egypt reported its first case of COVID-19 on 14th of February 2020 [2]. Egyptian Ministry of Health and Population attribute underestimation of the total number of patients with COVID-19 and an overestimation of the fatality rate to absence of open screening [3]. We hypothesized that physicians working during COVID-19 pandemic are more susceptible to develop different types of headache. Physicians are mandated to wear personal protective equipment (PPE) for a long time. Previous reports show that there was relative high prevalence of headaches with the use of the PPE especially N95 face‐mask amongst healthcare providers working in high‐risk areas [4]. This may be related to mechanical factors (external compression), hypoxemia, hypercapnia or the stress associated with its use [5]. Additionally, wearing close-fitting face mask can lead to compression on superficial nerves in the neck which may aggravate underlying cervical neck strain which lead to face‐mask‐associated headaches especially cervicogenic headache [6, 7]. Another recent study done in Singapore who evaluated the prevalence and characteristics of de novo headaches associates with PPE usage among healthcare providers during COVID-19 pandemic show that about 81% of the participants developed de novo PPE‐associated headaches [8]. They stated that most of their healthcare providers developed de novo PPE‐associated headaches or worsening of their pre‐existing headache disorders [8]. We aimed in this study to determine prevalence of new onset headache among physicians working in COVID-19 isolation hospitals and it’s impact on their performance. We also aimed to explore factors affecting their headache and it’s clinical characteristics.

## Methods

This study was a cross sectional study performed at Ain Shams university hospitals and Saudi German Hospital, both hospitals had a COVID-19 area including emergency department, intensive care units and isolation wards as well as out-patient clinics and outpatient services for non COVID-19 patients. The study was conducted in June 2020, 4 months after the COVID-19 outbreak in Egypt. Participants were included if they were physicians. Participants were excluded if they had prior COVID-19 infection. Written informed consent was obtained from all participants and the study was approved by the ethical committee of Ain Shams University. The questionnaire was sent to all of the physicians on duty in both hospitals on their e-mails during the study period.

All of those who agreed to participate completed a self‐administered questionnaire. The questionnaire was divided into sections: First section included demographics (age, gender, position whether specialist or consultant, department, working area whether COVID-19 area or non COVID-19 area, past history), working hours and PPE usage (hours of work per week, sleeping hours per day, type of PPE used, hours of PPE usage per week for each type). Headache was classified according to International Classification of Headache Disorders, 3rd Edition (ICHD-3) (2018) [9]. Section 2 included analysis of pre-existing headache (type of headache, character of headache, site, tender points, frequency, duration of headache and intensity of headache using visual analog scale (VAS) [10] before and after COVID-19 outbreak, abortive and prophylactic medications used, compliance to medications and causes of non-compliance if applicable, triggers of headache, how much is the headache impairing function on a scale from 1 to 5, how much is it affecting encounters with patients, using computers at work, days of absence due to headache, need to take abortive medications during or after the shift, being reluctant to wear the PPE due to the headache). Section 3 included analysis of new onset headache (type of headache, character of headache, site, tender points, frequency, duration and intensity of headache, triggers of headache, how much is the headache impairing function on a scale from 1 to 5, how much is it affecting encounters with patients, using computers at work, days of absence due to headache, need to take abortive medications during or after the shift, being reluctant to wear the PPE due to the headache).

*Statistical analysis* Sample size was calculated using PASS program version 15, setting the type-1 error (α) at 0.05 and the margin of error at 7%. Result from previous study [8] showed that out of 158 health workers, 128 (81.0%) respondents developed headaches during COVID-19. Calculation according to these values produced a minimal sample size of 160 respondents taking in account dropout rate and incomplete questionnaires.

Data were revised, coded, entered on a computer and analyzed using SPSS package version number 20. Quantitative data were tested for normality with Shapiro–Wilk test and described as mean, standard deviation (SD) or median/ interquartile range according to data distribution. Student *t*-test and Mann Whitney were used for comparing quantitative variables between two study group Qualitative data were expressed as frequencies (*n*) and percentage (%). Chi-square was used to test the association between qualitative variables. Multivariate logistic regression analysis was performed for finding the predictors of suffering new form of headache. Variables found to be significant in univariate analysis were included in the multivariate analysis model and ROC curve. *P*-value ≤ 0.05 was considered significant.

## Results

**Fig. 1:**
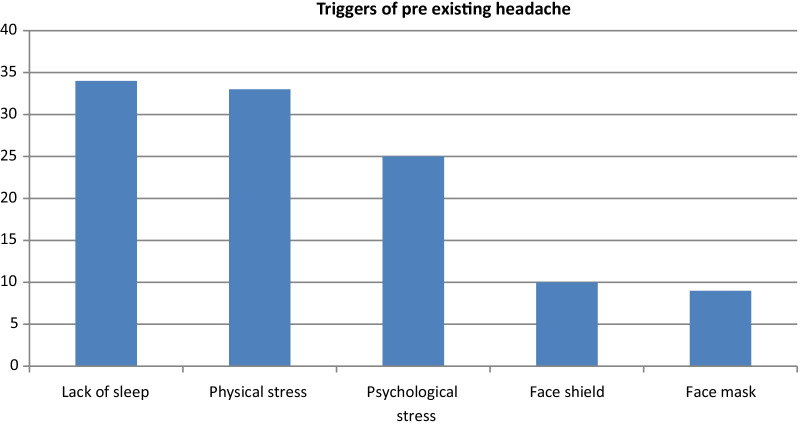
Triggers of pre-existing headache

**Fig. 2:**
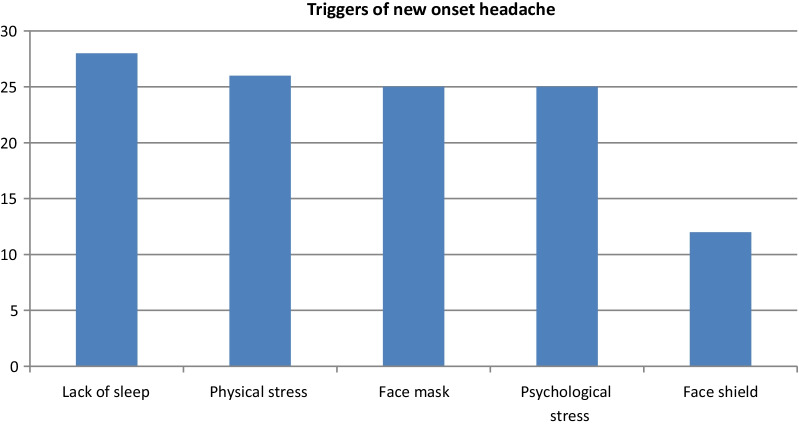
Triggers of new onset headache

The questionnaire was sent to 200 physicians, 185 responded giving a response rate of 92.5%, 20 were excluded for having history of prior COVID-19 infection, The majority of our participants were males 114 (69.1%) versus 51 (30.9%) females, age mean was 33.49 ± 5.37. Past medical history was found in 22 participants (13.3%) (Table 1), 14 (8.5%) were smokers, 136 (82.4%) were specialists and 29 (17.6%) were consultants in different departments (Table 2), 140 (84.8%) worked in COVID-19/isolation area, while 25 (15.2%) worked in outpatient services. The mean of sleeping hours among participants was 6.38 ± 0.98 h/day with a range of 4–8 h, the mean of working hours was 52.62 ± 17.96 h/week with a range of 8–96 h. The pattern of PPE usage among participants showed that the most commonly used mask was surgical mask 84 (50.9%) followed by N95 mask 55 (33.3%) followed by full face respirator 26 (15.8%), while the most commonly used protective eye wear was face shield 54 (32.7%) followed by goggles 29 (17.6%), while 83 (50.3%) of the participants accounted on combined PPE usage. Wearing face mask showed a mean of 44.26 ± 19.86 h/week (8.77 ± 3.21/day). While wearing protective eye-wear showed a mean of 33.05 ± 22.26 h/week (6.89 ± 3.92/day), while the duration for combined wear of face mask and protective eye wear showed a mean of 5.92 ± 3.53 h/day. Our study showed that 76 (46.1%) participants did not experience any headache, 51 (30.9%) had pre-existing headache prior to the COVID-19 outbreak, while 38 (23%) experienced new onset headache that was not experienced before the COVID-19 outbreak (Table 3). Among our participants 51 (30.9%) had pre-existing headache that started prior to the COVID-19 pandemic, the duration of headache had a mean of 7.47 ± 7.55 h per day, the intensity of the previous headache showed a mean of 6.45 ± 1.95 h per day. The most commonly encountered headache was migraine in 31 (60.8%) followed by tension headache in 16 (31.4%) followed by cluster headache 4 (7.8%). The most common character of pain was throbbing pain 29 (56.9%), followed by dull aching pain 11 (21.6%) followed by pressure tight band headache 8 (15.7%) followed by stabbing pain 3 (5.9%). Site of headache and presence of tender points was accounted for as shown in Table 4. Frequency of headache was less than 15 days/month in 42 (82.4%) and more than 15 days/month in 9 (17.6%). Eleven (21.6%) accounted on non-compliance on their maintenance medications due to being not effective anymore in 5 (45.5%), difficulty to obtain them due to the COVID-19 situation in 2 (18.2%) and due to forgetting to take the medications during the shift in 4 (36.3%). Among the 51 participants complaining from pre-existing headache, 42 (82.4%) accounted on worsening of their usual headache in the form of increase either in frequency, duration or intensity. While 10(19.6%) accounted on headache different in site and character than their usual headache describing a pressure tight band headache at the back of the head 5 (50%) or on the temple and forehead 5 (10%). Participants were asked about what they feel triggers their headache the most (allowing more than one choice) (Fig. 1). Among our study group 38 (23%) participants accounted on experiencing new onset headache that was never experienced before. The character of that headache was described as pressure tight band headache in 18 (47.4%), dull aching in 13 (34.2%), throbbing in 6 (15.8%) and stabbing in 1 (2.6%). Site of headache and presence of tender points was accounted for as shown in Table 5. Frequency of headache was less than 15 days/month in 28 (77.8%) participants and more than 15 days/month in 8 (22.2%) participants. The intensity of headache via VAS showed a mean of 5.34 ± 1.44, while duration of the headache showed a mean of 3.46 ± 3.99 h per day. Participants were asked about what they feel triggers their headache the most (allowing more than one choice) (Fig. 2).

**Table 1:** Past medical history of study participants

	*n*	%
Past medical history		
Hypertension	11	6.7%
Diabetes	5	3.0%
Asthmatic	3	1.8%
Multiple Sclerosis	1	0.6%
Ischemic heart disease	1	0.6%
Hodgkins lymphoma	1	0.6%

**Table 2:** Specialties of study participants

Department	*n*	%
Anesthesia and Intensive care unit	37	22.4%
Emergency medicine	33	20.0%
Internal medicine	26	15.8%
General surgery	10	6.1%
Neurosurgery	7	4.2%
Cardiology	7	4.2%
Psychiatry	6	3.6%
Neurology	6	3.6%
Pediatrics	5	3.0%
Vascular Surgery	4	2.4%
Radiology	4	2.4%
Ophthalmology	4	2.4%
Orthopedic	3	1.8%
Plastic surgery	2	1.2%
Oncology	2	1.2%
Obstetrics and Gynecology	2	1.2%
Chest	2	1.2%
Cardiothoracic	2	1.2%
Urology	1	0.6%
Otorhinolaryngology	1	0.6%
Dentistry	1	0.6%

**Table 3:** Headache location

	*n*	%
Site of headache		
Right/left Side	22	43.1%
Behind eye	13	25.5%
Forehead	11	21.6%
Back	8	15.7%
Allover	8	15.7%
Vertex	5	9.8%
Temple	3	5.9%
Pressure pain or tenderness		
None	15	29.4%
Supra-orbital	16	31.4%
Back of head	15	29.4%
Vertex	3	5.9%
Shoulders	1	2.0%
Facial	1	2.0%

**Table 4:** Pain and trigger sites among participants

	*n*	%
Site of pain		
Forehead	17	44.70%
Behind eye	10	26.30%
Allover	9	23.70%
Back	6	15.80%
Vertex	6	15.80%
Temple	3	7.90%
Right/left Side of head	2	5.30%
Pressure pain or tenderness		
None	10	26.3%
Back of head	9	23.7%
Supra-orbital	7	18.4%
Vertex	5	13.2%
Infraorbital	5	13.2%
Shoulders	2	5.2%

**Table 5:** Comparison between physicians with and without out new onset headache (including those with newly altered headache) as regard working hours and PPE usage characteristics

		New onset and change of pre-existing headache				*P*	Sig
		No		Yes			
		Mean	± SD	Mean	± SD		
Duration facial mask and protective eye-wear are worn together (hours)		5.52	3.98	6.07	3.37	0.4**	NS
PPE face mask	Surgical mask	46	54.8%	38	45.2%	0.17^‡^	NS
	N95 mask	30	54.5%	25	45.5%		
	Full face respirator	9	34.6%	17	65.4%		
PPE eye wear	None	64	78.0%	18	22.0%	0.001^‡^	HS
	Goggles	13	44.8%	16	55.2%		
	Face Shield	8	14.8%	46	85.2%		
Combined face and eye PPE usage	No	64	78.0%	18	22.0%	0.001^‡^	HS
	Yes	21	25.3%	62	74.7%		

*PPE* personal protective equipment, *NS* non-significant, *HS* highly significant

*Student *t* test

**Mann Whitney test

^‡^Chi-square tests

Impact of headache on function was assessed among participants with headache (89 participants), On a scale from 1 to 5 the median was 3 (2–4), the majority of participants 49 (55.06%) accounted that the headache forced them to interrupt their encounter with patients “sometimes”, while 26 (29.21%) accounted that this “rarely” happened, 12 (13.48%) said it “never” happened and 2 (2.25%) said it occurred “most of the time”. The majority of participants 42 (47.29%) also accounted that the headache affected their use of computers at work “sometimes”, while 26 (29.21%) accounted that this “rarely” happened, 15 (16.85%) said it occurred “most of the time” and 6 (6.74%) mentioned that it “never” happened. Regarding the need to take analgesics during the shift 41 (46.07%) accounted that they needed to take analgesics “sometimes” during the shift, 25 (28.09%) needed them “most of the time” while 15 (16.85%) “rarely” needed them and 8 (8.99%) never needed them. Eighteen participants (20.22%) needed to take days off from work due to the headache ranging from 1 to 6 days.

There was no statistically significant difference between physicians with new onset or change of pre-existing headache and physicians without new onset or change of pre-existing headache as regard socio-demographic characteristics except for gender, where 68.6% of females had headache compared to 39.5% only among males. There was no statistically significant difference between physicians with new onset or change of pre-existing headache and physicians without new onset or change of pre-existing headache as regard working hours and PPE usage characteristics except for PPE eyewear, and Combined Face and eye PPE usage. For PPE eyewear, 46 (85.2%) of physicians wearing face shield had headache compared to 16 (55.2%) and 18 (22%) of those wearing goggle and non-users respectively. Similarly, 62 (74.7%) of physicians wearing both face and eye PPE had headache compared to 18 (22%) only of non-users. Using multivariable logistic regression, it was shown that females were at higher risk of experiencing headache (odds ratio [AOR] 3.31; 95% CI 1.4–7.8; *P* = 0.006), compared to males. Participants using Combined Face and eye PPE usage were at higher risk of developing headache compared to single PPE users (AOR 19.8; 95% CI 7.7–28.8; *P* = 0.001), Participants wearing face shield were at higher risk of developing headache compared to eyewear non users (AOR 15.8; 95% CI 1.63–23.7; *P* = 0.017).

## Discussion

Our study aimed to determine prevalence of new onset headache among physicians from different specialties working in COVID-19 isolation hospitals and it’s impact on their performance and function with a special focus on the clinical characteristics and factors affecting their headache. Among our participants 48.5% developed headache (either new-onset headache or change in previously existing headache). Agreeing with another study we found that about half of respondents who developed their headache during working in COVID-19 hospitals described pressure tight band which is one of the common diagnostic criteria for tension type headache. Although the actual underlying etiology of tension headache is unknown yet activation of peripheral hyperexcitable peripheral afferent neurons from neck muscles is the most likely explanation for this type of headache [11]. Our hypothesis was that working under stress and long shifts during this pandemic are the triggers of this type of headache. To our knowledge, this study is considered pioneer regarding reviewing different factors that may predispose to headache during COVID-19 pandemic among physicians specifically. Our study found that PPE eyewear and combined face and eye PPE usage are the most important predisposing factors of new onset headache among respondents. This was in agreement with a recent study done in Singapore during this global pandemic. They found that the combined exposure to N95 face mask and protective eyewear use for more than 4 h/day predisposed to a greater likelihood of developing de novo headache [8]. Our results also goes with previous reports which stated that face mask especially N95 is related to new onset headache in healthcare providers [12, 14]. This can be attributed to multiple factors such as mechanical compression, hypoxia and rebreathing of carbon dioxide. It was also suggested that the traction forces induces irritation to the superficial sensory nerves as well as the activation of the trigeminocervical complex which induces nociceptive pain through transmission to the higher brain centers [13]. We tried in this study to find independent factor/s that attribute to new onset headache among our sample of physicians. We found that participants using combined face and eye PPE were at higher risk of developing headache compared to single PPE users. Additionally, our results explored that face shield users are at higher risk of developing headache compared to eyewear non-users. This may be attributed to the mechanical pressure effect of those shields over the forehead and occipital region that may lead to external compression headache. The mechanism responsible of this type of headache is that the compression of different sensory branches of trigeminal nerve in the face and compression of occipital nerve [14]. The peripheral sensitization of those sensory branches can lead to activation of trigemino-cervical complex through nociceptive information and then transmitted through trigeminal ganglion and trigeminal nuclei in brain stem to cortical areas that trigger headache attacks [15]. Previous reports suggest that the external pressure may lead to worsening of migrainous headache in predisposed patients especially if stimulus is prolonged [14]. This hypothesis is supported by reports from our responders during the survey about the pain and tenderness in different pressure sites. Approximately 24% of our sample described pressure pain in occipital region, 18% in supraorbital region, 13% in infraorbital region and vertex.

Surprisingly, we did not find a statistically significant difference between physicians with and without new onset headache (including those with newly altered headache) as regard working hours. This was different from recent study done in Singapore which stated that prolonged working (more than 4 h per day) with combined use of N95 face mask and eyewear was independently associated with developing de novo PPE‐associated headaches [8]. Our survey explore that the respondents refer worsening of their headache to other triggers like sleep deprivation, physical and psychological stress. This finding is supported by previous reports which concluded that sleep deprivation and fatigue are common precipitating factors for migraine and tension type headache [16]. Our analyses indicated that a significant proportion of the participants had mild to moderate functional impairment. Sixty percent of the participants reported that their headache sometimes interrupt their work. That finding raises the importance of proper management of headache which has direct impact on productivity of physicians during COVID-19 pandemic. We have some limitations of our study. First, sample size is considered small but this was attributed to difficult accessibility to physicians working during these circumstances inside isolation hospitals and study was conducted early at the beginning of the pandemic in a short time period because we aimed to assess physicians before being exposed to COVID-infection. Second, we did not include other healthcare providers as nurses in our study because of the language barrier (as our self-administered questionnaire was in English). Third, we did not assess the response to medications.

## Conclusion

We discovered that about 48.5% of physicians working in COVID-19 hospitals developed either new-onset headache or accounted on change in their previously existing headache**.** This study explored that PPE eyewear and combined face and eye PPE usage are the most important predisposing factors of new onset headache among respondents**.** Most of studied physicians reported that their headache sometimes interrupted their work.

## Data Availability

All raw data will be available on the editor request through communication with the corresponding author.

## Abbreviations

COVID-19: Coronavirus disease-19
PPE: Personal protective equipment
VAS: Visual analog scale
WHO: World Health Organization
